# Recombination within the *Cepaea nemoralis* supergene is confounded by incomplete penetrance and epistasis

**DOI:** 10.1038/s41437-019-0190-6

**Published:** 2019-02-14

**Authors:** Daniel Ramos Gonzalez, Amaia Caro Aramendia, Angus Davison

**Affiliations:** 1 School of Life Sciences, University of Nottingham, Nottingham, NG7 2RD UK; 2 Departamento de Zoología y Biología Celular Animal, Universidad del País Vasco, Paseo Univ 7, Vitoria, 01006 Spain

**Keywords:** Evolutionary biology, Evolutionary genetics

## Abstract

Although the land snail *Cepaea nemoralis* is one of the most thoroughly investigated colour polymorphic species, there have been few recent studies on the inheritance of the shell traits. Previously, it has been shown that the shell polymorphism is controlled by a series of nine or more loci, of which five make a single ‘supergene’ containing tightly linked colour and banding loci and more loosely linked pigmentation, spread band and punctate loci. However, one limitation of earlier work was that putative instances of recombination between loci within the supergene were not easily verified. We therefore generated a new set of *C. nemoralis* crosses that segregate for colour, banding and pigmentation, and several other unlinked shell phenotype loci. The snails were genotyped using a set of RAD-seq-derived loci that flank the supergene, and instances of recombination tested by comparing inferred supergene genotype against RAD-marker genotype. We found no evidence that suspected ‘recombinant’ individuals are recombinant between loci within the supergene. As point estimates of recombination between both colour/banding, and colour/pigmentation loci are zero, incomplete penetrance and epistasis are a better explanation for the apparent ‘recombinant’ phenotype of some snail shells. Overall, this work, therefore, shows that the architecture of the supergene may not be as previously supposed. It also provides a resource for fine mapping of the supergene and other major shell phenotype loci.

## Introduction

Historically, some of the most important animals in studying colour polymorphism have been the land snails *Cepaea nemoralis* and the sister taxon, *C. hortensis*, because it is straightforward to collect them and record the frequencies of the different morphs in different locations and habitats (Cain and Sheppard 1950; Cain and Sheppard 1952; Cain and Sheppard 1954; Jones et al. 1977). There is also the benefit that the major loci that determine the polymorphism show simple Mendelian inheritance (Cook 1967; Jones et al. 1977). However, while ongoing and long-term studies on these animals continue to provide compelling evidence for the fundamental role of natural selection in promoting and maintaining variation in natural populations, as well as the impact of modern-day habitat change (Cameron and Cook 2012; Cook 2017; Silvertown et al. 2011), the last research on the inheritance of the loci that determine the polymorphism dates to the late 1960s. This is a problem because now that there is finally some progress towards identifying the genes involved (Kerkvliet et al. 2017; Mann and Jackson 2014; Richards et al. 2013), it is important that laboratory crosses are available, to validate prior knowledge on the inheritance and for use in fine mapping recombination break-points.

Previous work has shown that the shell polymorphism is controlled by a series of nine or more loci, of which five or more make a single ‘supergene’, containing linked shell ground colour (*C*), banding (*B*), band/lip pigmentation (*P*/*L*), spread band (*S*) and punctate (or ‘interrupted’; *I*) loci. In most studies, colour and banding have been found to be tightly linked, with recombination typically towards the lower end of 0–2% (Cain et al. 1960; Cook 1967; Cook and King 1966). The exceptions are a study by Fisher and Diver (1934), which reported recombination of ~20% between *C/B*, and two crosses in Cain et al. (1960) which showed recombination of ~16%, also between *C/B*. Although there have been fewer studies, pigmentation, spread band and punctate are believed more loosely linked, showing rates of recombination between 3 and 15% (Cain et al. 1960; Cain et al. 1968; Cook 1967). The main other loci that make up the shell phenotype are various forms of band-suppressing loci, all unlinked to the supergene, including the mid-band locus, *U* (unifasciata), and another that suppresses the first two bands, *T* (trifasciata).

One unavoidable limitation of prior works was that putative instances of recombination between loci within the supergene could not be verified, except by breeding further generations of snails from the ‘recombinant’ offspring to confirm the underlying genotype. This was rarely possible, perhaps due to logistics combined with the fact that many pairs do not produce offspring. Nonetheless, it was recognised that incomplete penetrance might be an alternative explanation for the phenotype of recombinants. Chance arrangements of alleles at other loci might sometimes interact to prevent expression of a particular phenotype, causing individuals to appear as if they are ‘recombinant’ (Cook and King 1966).

To further understand the frequency of recombination within the supergene, and to generate further material for fine mapping, we made a new set of *C. nemoralis* crosses that segregate for several shell phenotype loci. The offspring were then genotyped using a set of linked RAD-seq loci that flank either side of the supergene (Richards et al. 2013), and instances of recombination confirmed or refuted by comparing inferred supergene genotype against RAD-marker genotype. The underlying idea is that individuals that show recombination within the supergene should also be recombinant by RAD-marker. Overall, we found that the phenotype of ‘recombinant’ individuals is better explained by incomplete penetrance and epistasis.

This work, therefore, provides a method to identify recombination events that either flank the supergene or are between loci within the supergene. The results also show that recombination within the supergene may be considerably rarer than supposed.

## Materials and methods

### The culture of *Cepaea*

Snails were fed a hydrated grass pellet, oat and chalk mix, supplemented with lettuce, as described previously (Davison 2000). Generally, large juvenile virgin snails were raised to adulthood in isolation and then introduced to a partner. Pairs of snails were then kept in tanks with ~4 cm soil until egg laying began. As *C. nemoralis* is a simultaneous hermaphrodite, offspring from both parents were used. Egg batches were isolated, and the offspring reared to adulthood under the same feeding regime, with the time from egg to adult being ~6 months. Parents and adult offspring, or large subadult offspring were preserved frozen.

The majority of snails were raised to adulthood, and so the colour, banding, band pigmentation and lip colour phenotype was scored and then the shell genotype inferred. Complications in scoring some characters necessitated minor deviations from the scheme put forward by Cain (1988), summarised in Table 1. To distinguish phenotype from genes and genotypes, symbols for the latter are in italics.

**Table 1:** Phenotypes and genotypes of shell characters used in this study

Phenotype				Genotype		
Character	Description	Notation		Locus	Allele	
Ground colour	Brown		B	Ground colour	brown	*C* ^*B*^
	Pink		P		pink	*C* ^*P*^
	Yellow		Y		yellow	*C* ^*Y*^
Banding	Unbanded	00000	O	Banding	unbanded	*B* ^*O*^
	First two bands missing	00345			normal banded	*B* ^*B*^
	First band missing	02345		Band pigmentation	normal	*P* ^*N*^
	Mid-banded	00300	M		hyalozonate	*P* ^*H*^
	Banded	12345	B	Lip pigmentation	normal	*L* ^*L*^
	Spread-banding		S		white lip	*L* ^*A*^
				Spread-banding	spread	*S* ^*S*^
					normal	*S* ^*-*^
Band pigmentation	Normal pigmented bands		N	Mid-banding	mid-banded	*U* ^*3*^
	Unpigmented bands		H	(aka unifasciata)	normal banded	*U* ^*-*^
	(aka hyalozonate)			Trifasciata	first two bands missing	*T* ^*345*^
Lip pigmentation	Normal pigmented lip		L		normal banded	*T* ^*-*^
	White lip		A	Hypothesised	first band missing	*X* ^*2345*^
	(aka albolabiate)				normal banded	*X* ^*-*^

The ground colour, banding, band, spread band, and lip pigmentation loci are linked in a supergene. The other loci are unlinked. Alleles are shown in dominance order

We have previously shown that colour variation is multimodal but continuously variable in natural populations, necessitating the use of quantitative methods to measure it (Davison et al. 2018). However, in simple crosses it is straightforward to bin the individuals into one of two types; quantitative measures are not necessary. Therefore, the shell ground colour phenotype was scored as either yellow (Y), pink (P) or brown (B), and where possible, the corresponding genotype inferred (dominance is *C*^*B*^ > *C*^*P*^ > *C*^*Y*^), including whether in coupling or repulsion phase with other loci (Table 1).

Similarly, banding phenotype was generally scored as either unbanded (O; 00000), mid-banded (M; 00300) or having several bands (B; most frequently 12345, but all combinations except 00300) and the genotype at the banding locus was inferred (unbanded dominant; *B*^*O*^ > *B*^*B*^). Several crosses also segregated for the mid-band locus, so the genotype at that locus was also inferred (mid-band dominant; *U*^*3*^ > *U*^*-*^). One cross potentially segregated for two other band-suppressing loci, *T* (first two bands missing: 00345; *T*^345^ > *T*^-^) and another possible locus, which we called *X* (first band missing or very faint: 02345; X^2345^ > X^-^).

It is not clear from previous studies as to whether the lip pigmentation is a separate locus, or is instead allelomorphic with the band pigmentation locus. As a precaution, the two were therefore treated separately. Thus, band pigmentation phenotype was scored as normal (N) or hyalozonate (H) and the corresponding genotype inferred (*P*^*N*^ > *P*^*H*^). Hyalozonate shells typically have unpigmented bands and lip (see Discussion); discrete bands can still be recognised because the background colour is paler than the shell ground colour. In some crosses, the lip pigmentation phenotype was two distinct types, either normal (L), or white lip (A; albolabiate), and so the corresponding genotype was inferred (*L*^*L*^ > *L*^*A*^). In other crosses, lip pigmentation showed quantitative variation and so was difficult to score. One cross also showed variation in spread-banding, another locus of the supergene, for which the spread band allele, *S*^*S*^, is dominant to normal banding, *S*^*-*^.

Some of the adults used were wild-collected from either the UK, Ireland or Spain; others were derived from prior laboratory crosses (Table 2). The adults used in crosses 10, 11, 12 and 13 were derived from offspring of cross 9, so the shell genotype could be inferred with extra confidence. This was aided by full-sib inbreeding in producing crosses 10, 11 and 12, and another round of inbreeding to produce cross 13.

**Table 2:** Summary of parent and offspring phenotypes from *C. nemoralis* crosses

		Parent		Source		Offspring								Linked loci						Unlinked			Putative	Chi-squared			
Cross		phenotype				number	phenotype							*C*	*B*	*L*	*P*	*S*		*U*	*T*	*X*	recombinant?	*p* value			
1		P O	Y M	Wye Valley, Derbyshire	Marlborough Downs, Wiltshire/ Slieve Carron, Ireland		P M	Y O																			
		C100	C101			103	56	47						103	103									0.375	0.375		
2		P O	Y M	Marlborough Downs, Wiltshire/ Slieve Carron, Ireland	Marlborough Downs, Wiltshire/ Slieve Carron, Ireland		P O	Y M	**Y O**																		
		C102	C103			43	20	20	**3**					43	43								*C/B*	0.647	0.647		
3		P O	Y M	Marlborough Downs, Wiltshire/ Slieve Carron, Ireland	Marlborough Downs, Wiltshire/ Slieve Carron, Ireland		P O	Y M																			
		C104	C105			46	27	19						10	10									0.238	0.238		
4		P O	Y B	Nottingham	Esles, Spain		P B	Y O																			
		C110	C111			27	17	10						27	27									0.178	0.178		
5		P O	Y B (12345)	Nottingham	Esles, Spain		P B (00345)	P B (12345)	P B (02345)	Y O	**P M**															00345/banded	02345/banded
		C112	C113			109	27	12	16	53	**1**			109	109						55	28		0.847	0.847	0.893	0.450
6		P O	Y B	San Roque, Spain	Esles, Spain		P O	Y B	Y M																		
		C114	C115			34	18	8	8					34	34					17				0.732	0.732		
7		P M	Y M	Offspring of C101 × C102	Offspring of C104 × C105		P M	Y M																			
		C119	C118			75	37	38						75										0.908			
8		P O L	Y M L	San Roque, Spain	San Roque, Spain		P M L	Y O L	Y O A	**P O L**																albolabiate	
		C108	C109			50	26	11	12	**1**				50	50	25							*C/B*	0.572	0.777	0.870	
9		P M N	Y B H	Offspring of C108 × C109	Nottingham		P M N	P B N	Y M N	Y B N																	
		C116	C120			16	4	4	3	5				16						16				1.000	0.617		
10	Inbreeding	P M N	Y B N	Offspring of C116 × C120	Offspring of C116 × C120		P M N	P B N	Y M N	Y B N	Y M H	Y B H															
		C450	C449			12	5	2	2	1	2	0		12			6			12				0.564	0.083	0.505	
11	Inbreeding	P M N	Y B N	Offspring of C116 × C120	Offspring of C116 × C120		P M N	P B N	Y M N	Y B N	Y M H	Y B H	**P M H**														
		C451	C452			116	34	28	12	22	10	6	**4**	116			28			116			*C/P*	0.137	0.710	0.054	
12	Inbreeding	P M N	Y B N	Offspring of C116 × C120	Offspring of C116 × C120		P M N	P B N	Y M N	Y B N	Y M H	Y B H	**P M H**													hyalozonate	
		C662	C665			146	39	46	7	19	15	12	**8**	146			73			146			*C/P*	0.001	0.508	0.774	
13	Inbreeding	P M N	Y B H	Offspring of C451 × C452	Offspring of C662 × C665		P M N	P B N	Y M N	Y B N	Y M H	Y B H	**P M H**													hyalozonate	
		C825	C841			63	14	18	0	0	20	9	**2**	63			63			63			*C/P*	0.529	0.257	0.900	
14		Y B S	Y O	UK	UK		Y B S	Y O																			
		C568	C569			44	28	16							44			44					Not informative				
Total						884								804	420	25	170	44	0	370	55	28					

Phenotypes that may be due to a recombination event in a parent are highlighted in bold. Inferred genotypes of offspring are detailed in Supplementary Table 1. Key: P pink, Y yellow, O unbanded, M mid-banded, B all other banding patterns; N normal band pigmentation; H hyalozonate banding (nearly always with white lip—see text); S spread-banding; L normal lip pigmentation; A albolabiate (white lip). Cross 5 also showed segregation for another one or two band-suppressing loci, T and X, so the detailed banding notation is also shown

For each cross, Mendelian segregation ratios were tested using chi‐square goodness‐of‐fit tests.

### DNA extractions and RAD-marker genotyping of parents and offspring

For future mapping of the supergene and other shell-character loci, we wished to identify individuals that show evidence of recombination, ideally either close to a shell-character locus, or between loci within the supergene. As we have previously isolated RAD-seq markers that flank the supergene (Richards et al. 2013), we could use those markers to confirm or refute individuals that show a phenotype that might have arisen due to recombination within the supergene.

To this end, genomic DNA was extracted from frozen snail tissue, as described previously (Richards et al. 2013), using foot because it is a good source of high molecular weight DNA. For most samples, slices of snail tissue were incubated at 65 °C in extraction solution (3% CTAB, 100 mM Tris‐HCl, pH 7.5, 25 mM EDTA, pH 8, 2 M NaCl) with 0.2 mg/mL proteinase K and 80 μg/mL RNase. Upon lysis, a chloroform extraction was performed, then three volumes of CTAB dilution solution added (1% CTAB, 50 mm Tris‐HCl, pH 7.5, 10 mM EDTA, pH 8). Samples were mixed until a precipitate appeared, then the supernatant removed. The pellet was washed twice in 0.4 M NaCl in TE (0.4 M NaCl, 10 mM Tris‐HCl, pH 7.5, 1 mm EDTA, pH 8), re-dissolved in 1.42 M NaCl in TE (1.42 M NaCl, 10 mM Tris‐HCl, pH 7.5, 1 mM EDTA, pH 8), then precipitated in ethanol, centrifuged and dried. Latterly, a few samples were lysed in lysis buffer (10 mM Tris, 0.1 M EDTA, 0.5% SDS), then extracted using the standard phenol-chloroform protocol.

Then, a subset of the individuals in several of the crosses were genotyped using custom assays derived from RAD-seq loci that flank either side of the supergene. For this, standard PCR was carried out either using Amplitaq Gold polymerase (Invitrogen), 1.5 mM MgCl_2_, using a cycle of 95 °C for 10 min, followed by 35 cycles of 95 °C for 30 s, 58 °C for 30 s, and 72 °C for 1 min or Clontech Advantage 2 PCR with an initial denaturation of 95 °C for 1 min, followed by 35 cycles of 95 °C for 15 s, 65 °C for 1 min, 68 °C for 1 min, and 72 °C for 1 min. Primers and custom genotyping assays were based on the previously characterised RAD-seq loci (Richards et al. 2013), and varied according to the cross. The primers were RAD06F 5’-GCCTATCCGTCATTGTTGGT-3’ RAD06R 5’-GTCAAGGCTTGCTTCTTTGG-3’, RAD9F 5’-TTTCTCGGAACGACGGAGT-3’, RAD9R 5’-GGTCTCGTCAATGGCACTTT-3’, RAD11F 5′-AAGAAGCGTCCTTCTGGAAA-3’, RAD11R 5’-CACCTTCCCCATTCTTCAAA-3’. For each cross it was necessary to custom design an assay, so that segregation of markers could be observed in the offspring. The enzymes used with each assay and each cross are detailed in Supplementary Table 1.

## Results

### Segregation of Mendelian loci that determine shell phenotype

Shell colour, locus *C*, showed segregation in crosses 1–13 (Table 2; Supplementary Table 2), only deviating significantly from expected Mendelian segregation ratios in cross 12, with fewer yellow shells than expected.

Crosses 1–6 and 8 showed segregation for the band presence/absence locus, *B*, with no deviations from expected Mendelian ratios.

Crosses 6 and 9–13 showed segregating variation for the mid-band phenotype, coded by the unlinked *U* locus. The observed phenotype frequencies did not differ from the expected frequencies.

Crosses 10 to 13 showed segregation for the pigmentation (hyalozonate; *P*) locus, with no deviations from expected Mendelian ratios.

Cross 8 showed segregation for the putative lip colour locus, *L*. The offspring phenotype frequencies would be consistent with single locus, assuming that *L* is part of the supergene and treating lip colour phenotypes as either normal (N) or albolabiate lip (A); there was no deviation from expected Mendelian ratios. Offspring in several of the other crosses, especially 10–13, showed considerable and apparently continuous variation in lip colour. We, therefore, tried to score the lip phenotype in the conventional manner, having a phenotype as normal, pale, or albolabiate, and reconcile this with a knowledge of the parental phenotypes and genotypes (parents in crosses 8 and 9). No scheme that we devised fitted a simple Mendelian model. This fits with previous studies (Cain et al. 1968; Cook 2003). Unfortunately, it was not possible to quantitatively measure the lip colour, as we have done for shell ground colour (Davison et al. 2018), because the coloured part of the lip was frequently too small and also on a curved surface.

Cross 14 showed segregating variation in the spread band phenotype. However, as one parent was homozygous for the dominant spread band allele *S*^*S*^, the cross was non-informative for recombination with other supergene loci.

Finally, cross 5 showed segregating variation for the locus that suppresses the first two bands, converting a five-banded snail (12345) to three-banded (00345). The offspring phenotype frequencies would be consistent with single locus, with *T*^*345*^ dominant to *T*^*-*^, with both parents being heterozygote, except that this would require both parents to have a *T*^*345*^ allele; apparently not possible because one of the parents is 12345. As five of the offspring with suppressed bands have 02345 phenotype and eleven a 0:345 (: = trace) phenotype, then the results are consistent with their being two band-supressing loci, one that causes the 00345 phenotype and another that causes the 02345/0:345 phenotype (see Supplementary Table 2 for inferred genotypes).

### Putative recombinants between colour, banding and lip and band pigmentation loci

Previously, the colour (P/Y) and banding (B/O) phenotype for six crosses (1, 4, 5, 6, 7, 8) and 398 offspring was reported, and the genotype of flanking RAD-seq loci reported for cross 1 (Richards et al. 2013). In this new work, we raised a further 486 offspring from eight more crosses, and genotyped six further crosses using flanking RAD-seq loci. The combined data set of parent and offspring phenotypes, alongside inferred genotypes, is presented here together, summarised in Table 2, and presented in full in Supplementary Table 2.

**Fig. 1:**
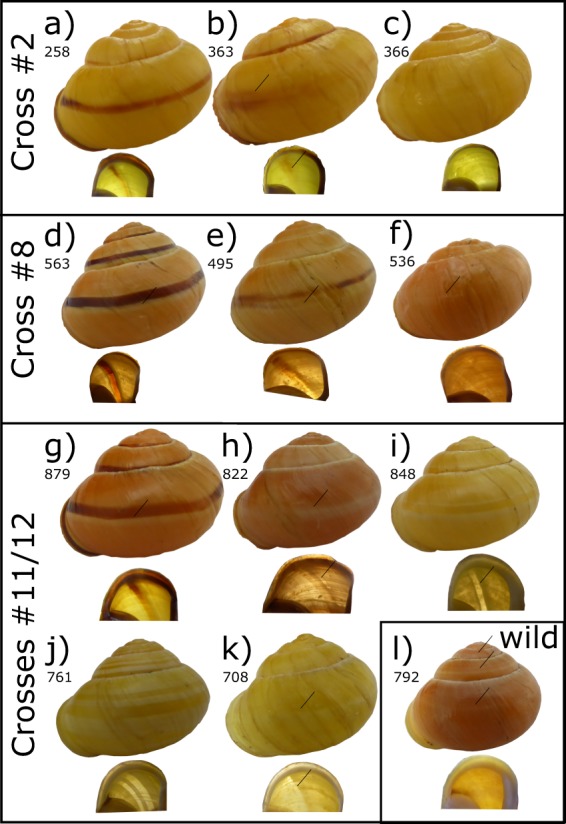
Shells of offspring from crosses, including putative recombinant individuals, and one wild collected individual. **a** Normal yellow mid-band, **b** yellow, trace of banding, **c** yellow, no band. An absence of banding suggests that individual 366 is a putative recombinant. **d** normal pink mid-band, showing evidence of white “highlighting” of pigmented band **e** pink, trace of banding, some highlighting **f** pink, no band, very faint mark where band would be. An absence of banding suggests that individual 536 is a putative recombinant. **g** Normal pink mid-banded, showing evidence of white highlighting of pigmented band **h** pink, no band, white highlighting **i** yellow, mid-band hyalozonate, **j** yellow, banded hyalozonate (02345) **k** yellow, mid-band hyalozonate. An absence of dark pigment suggests that 822 is a putative recombinant; however, the shell has retained the white highlighting pigment. Hyalozonate shells generally lack both dark and light pigment, see **i** and **j**; this is not always easily visible, see **k**. In a wild-collected pink hyalozonate, **l** the lack of pigment is just visible on some whorls, and not at all on some upper whorls, or from the inside

Offspring in several crosses produced snails with phenotypes that could be explained by a recombination event within the supergene of the heterozygous parent (Fig. 1).

Cross 2 produced three yellow unbanded snails (e.g., Fig. 1c), a phenotype that might be produced by recombination between the colour (*C*) and banding (*B*) loci; one of these individuals, a sub-adult with a damaged shell, has a very faint trace of a band. A few other snails in the same cross have much-reduced banding (e.g., Fig. 1b).

Cross 8 produced a single pink unbanded snail, a phenotype that is also best explained by recombination between the colour (*C*) and banding (*B*) loci (Fig. 1f). Very few of the snails in this cross have reduced banding. This cross also segregated for the lip pigmentation locus, *L*. As the recombinant snail has a pigmented lip (see Fig. 1f, lip image), then this cross in theory informs upon the order of loci within the supergene (but see below).

Cross 5 produced a single pink mid-banded snail. This phenotype is very difficult to explain by recombination, based on the known genotypes (Supplementary Table 1). As the pink colour of this snail is qualitatively different from the other pink-banded snails in this cross, the best explanation is that it is a likely a contaminant from another cross.

The remaining crosses produced offspring that suggest possible recombination between the colour *C* and band pigmentation *P* loci. Crosses 11, 12 and 13 produced several unbanded pink individuals, with pigmented lips (e.g., Fig. 1h). These were initially scored as hyalozonate, because the mid-band was evident but not pigmented. However, closer inspection revealed that the banding phenotype of these shells is not the same as the yellow hyalozonate shells. Specifically, the unbanded pink individuals retain the white highlighting pigment of a normal shell, but lack the dark pigment (compare Fig. 1h with 1g). This difference is especially evident when viewed from the underside: hyalozonate shells have cleared bands which are entirely lacking pigment whereas the white pigment of ‘unbanded’ snails shows a silhouette (compare Fig. 1h with 1i, j). Not all of the hyalozonate shells show such a clear pattern (e.g., Fig. 1k). For comparison, in a wild collected pink hyalozonate, the cleared bands are only evident on the upper whorls (Fig. 1l); in another shell they are not evident at all.

### Genotyping of offspring using RAD-seq derived loci

Individual offspring from crosses 1, 2, 8, 9, 10, 11, 12, and 13 were genotyped using custom assays derived from RAD-seq loci that flank either side of the supergene, using RAD06/RAD11 on one side and RAD09 on the other side (Table 3; Supplementary Table 2). Unfortunately, the RAD-seq loci in cross 5 lacked polymorphism so no assay was possible. To confirm or refute individual recombination events, we inspected the genotype of the putative recombinant offspring. In theory, individuals for which we have inferred recombination within the supergene should also show recombination by one of the RAD-markers.

**Table 3:** Summary of RAD-seq marker genotyping, putative number of supergene recombinants and actual number

Cross					Genotypes			Recombinants between supergene and:			Putative supergene recombinants	Actual supergene recombinants
Snails and phenotypes												
					RAD11	RAD06	RAD09	RAD11	RAD06	RAD09		
1	C100	C101	P O	Y M	101	102	102	1	10	1	0	0
2	C102	C103	P O	Y M	38		43	0		2	3	0
8	C108	C109	P O L	Y M L	44		50	2		1	1	0
9	C116	C120	P M N	Y B H		16	16		2	0	0	0
10	C450	C449	P M N	Y B N		12	12		2	1	0	0
11	C451	C452	P M N	Y B N	103		104		4	2	4	1?
12	C662	C665	P M N	Y B N	127		132		3	6	8	0
13	C825	C841	P M N	Y B H	44		49		4	1	3	0

RAD06 and RAD11 flank one side of the colour and banding loci of the supergene; RAD09 flanks the other side. Full genotypes are in Supplementary Table 1

None of the three individuals from cross 2 showed evidence of recombination from the flanking loci RAD11 and RAD09. Similarly, the single individual in cross 8 did not show evidence of recombination for the same RAD-seq loci. All eight putative recombinants in cross 12 and all three in cross 13 showed no evidence of recombination using RAD06 and RAD09; in cross 11, three individuals did not show evidence of recombination, with one single individual (804) showing recombination between RAD06 and the supergene and apparent recombination between the colour and pigmentation loci (*C*/*P*). If this were correct then it would inform the order of loci within the supergene. However, the phenotype of this snail is exactly the same as the other refuted recombinants (Fig. 1h). It is most likely not a hyalozonate, and therefore a coincidence that it also shows recombination between RAD06 and the supergene.

Thus, overall, while there is incomplete evidence in some cases, we were not able to confidently confirm any recombination events within the supergene. This puts the point estimate on recombination between *C* and *B* at 0/376 (<0.27%), between *C* and *P* at 0/170 (<0.60%) and between *C* and *L* at 0/25 (<4%). Therefore, the upper confidence limit for the mean rate of recombination, assuming that the probability of observing zero recombination events is 5%, is 0.80% for *C/B*, 1.76% for *C/P*, and 12.0% for *C*/*L*. Of course, as there are no recombination events, then this work does not inform the order of loci within the supergene; the higher upper limits for *C*/*P* and *C*/*L* are due to smaller sample sizes.

## Discussion

For future mapping and the precise identification of the supergene, it would be useful to identify individuals that are known to have a recombination break-point close to, or within the supergene. In this study, we initially identified four putative recombination events between the colour and banding loci (*C/B*) and fourteen putative recombination events between the colour and pigmentation loci (*C/P*). This is as expected because historic studies have indicated that *C/B* are more tightly linked than *C/P*. We also used genotyping of RAD-seq loci that flank the supergene in several of the crosses to reveal individuals that show recombination between a linked RAD-seq marker and the supergene. These RAD-seq markers may, therefore, be used for future recombination break-point mapping. However, the same RAD-seq genotyping falsified the putative inferences for recombination *within* the supergene. This is also supported by a close analysis of the phenotype of the shells, including variable penetrance of the mid-band phenotype (Fig. 1; crosses 2, 8, 11–13) and a comparison of true hyalozonate shells against shells that partially lack pigmentation (Fig. 1; crosses 8, 11–13).

Therefore, in contrast to previous studies that have reported rates of recombination between *C/B* of 0–2% and between *C*/*B* and the pigmentation locus, P, of 3–15%, we found zero recombinants, putting upper limits on the rate of recombination at 0.8 and 1.8%, respectively. This does not mean that previous inferences of recombination within the supergene were incorrect. However, as the ‘recombinants’ in this study are better explained by other means, then we would suggest that there is an absence of modern-day evidence for recombination within the *C. nemoralis* supergene. The structure of the supergene may not be as has previously been supposed.

### Incomplete penetrance and epistasis

Four individuals in crosses 2 and 8 were initially identified as putative recombinants, because they lacked the mid-band, or had only very faint traces of a band. If these snails had been true recombinant individuals then they would most likely be homozygous for colour and heterozygous for banding (genotype *B*^*0*^*B*^*b*^). Incomplete penetrance of the dominant *B*^*0*^ allele could mean that some individuals show evidence of banding (e.g., Fig. 1b, e). Instead, the genotyping shows that these individuals are not recombinant, and so must be genetically homozygous for colour and banding (*B*^*b*^*B*^*b*^). Therefore, the best explanation for their phenotype is that other loci are interacting epistatically to prevent full penetrance of homozygous banding alleles.

Similarly, up to fourteen individuals were identified that were putative recombinants between the colour and pigmentation loci. If they had been true recombinants, then they would most likely be heterozygous for colour and homozygous for pigmentation (*C*^*P*^*C*^*Y*^*P*^*H*^*P*^*H*^). However, close analysis of the phenotype and the genotyping together show that they are not recombinants and therefore, more likely they were heterozygous for both colour and pigmentation (*C*^*P*^*C*^*Y*^*P*^*N*^*P*^*H*^). These same snails are homozygous for the banding locus (*B*^*b*^*B*^*b*^), but segregate for mid-band phenotype (*U*^*3*^*U*^*-*^, *U*^*-*^*U*^*-*^); the putative recombinants were always mid-banded (*U*^*3*^*U*^*-*^), rather than fully banded (*U*^*-*^*U*^*-*^). The same explanation may apply, as above. Other loci sometimes interact to prevent full penetrance of the mid-banded phenotype.

The observation that the absence of a mid-band does not always have a simple genetic basis may shed some light on previous findings. For example, both Fisher and Diver (1934) and Cain et al. (1960) reported individual crosses that showed elevated rates of recombination between the *C*/*B* loci.

Cain et al. (1960) reported two crosses derived from the same mother that showed seven colour/banding recombinants in 43 snails, for which six were pink mid-banded snails. The expectation is that putative recombinants would have been homozygote for the banding locus (*C*^*P*^*C*^*Y*^*B*^*b*^*B*^*b*^) and non-recombinants heterozygous (*C*^*P*^*C*^*Y*^*B*^*0*^*B*^*b*^). However, if the six snails were not actually recombinants, then incomplete dominance of the band-suppressing allele (*B*^*0*^), or else epistatic interactions with other loci, may be an alternative explanation.

Similarly, Fisher and Diver (1934) described unexpectedly high recombination (20%) between colour and banding in one cross. Unfortunately, they did not report whether the snails used were mid-banded or not. However, in their specific case, doubt has been raised as to whether the individuals used were virgins before paired together (Cain et al. 1960; Ford 1971; Lamotte 1954). In researching this work, we were fortunate to find copies of letters between Fisher and Diver in the archive of Bryan Clarke (Supplementary Material). In letters from April/May 1934 that describe the preparation of the correspondence that was published in Nature in June 1934, there is clear admission that the snails used in the crosses were adult and not virgin. The authors partly acknowledge that this may be a problem. Referring to possible previous matings (“experience”), Fisher writes that "on fairly strong ground, which is not weakened by previous experience, but is not absolutely critical". Our interpretation of the text is that Fisher acknowledges that the snails may have previously mated, but discounts this as being a problem, because the offspring ratios approximate to that expected with limited recombination. There are therefore perhaps two errors, which together invalidate the conclusions of the published work.

Epistasis could also explain other earlier data on recombination between the *C*/*B* loci and the pigmentation locus. For example, in our study, we initially scored some pink individuals as hyalozonate, even though they had a lightly pigmented lip, only later realising our error. Other authors may have made the same mistake. Unfortunately, it is difficult to be certain from the previous literature whether pink hyalozonate recombinant individuals had an unpigmented lip, though if this was not the case then it might have been explicitly noted (e.g. p404 in Cook 1967). However, just as it is hypothesised that epistasis makes brown shelled individuals less likely to be banded, then it is reasonable to suppose that epistasis might mean that pink hyalozonates more rarely have a wholly unpigmented lip.

Evidently, further crosses are required, especially with respect to the other major loci, especially pigmentation, spread band and punctate loci in the supergene, and the various band-modifying genes. It is possible that some of these phenotypes, especially those for band-modification (Wolda 1969), are under multi-factorial control and/or dependent upon genetic background. A further general consideration is that the genetics may differ depending upon the location of origin of the snails. In our study, the snails are derived from the UK, Ireland or Spain, and hybrids between them. As a previous mitochondrial DNA study has shown that the *Cepaea* snails in the West of Ireland are at least partly derived from snails from the Pyrenees, and genetically divergent from those of the UK, then perhaps location should be more properly considered (Grindon and Davison 2013).

### Future progress

Overall, this work provides a resource for fine mapping of the supergene, and the other major shell phenotype loci. On the one hand, we have shown that phenocopies may be a problem in using the shell phenotype alone to detect recombination events within the supergene. On the other hand, the genotyping methods that we have introduced enable a means to avoid this problem.

Jones et al. (1977) (in)famously questioned whether understanding polymorphism in *Cepaea* is “a problem with too many solutions?” The intention of that work was to emphasise the perfect case study provided by *Cepaea*. We hope that these crosses may soon be used with new long-read DNA sequencing methods to assemble the *C. nemoralis* genome and to identify the supergene. Perhaps soon, polymorphism in *Cepaea* may instead be considered “a solution to many problems.”

### Data archiving

All relevant data is in the tables and Supplementary Material.

## Supplementary information

Supplementary Table 1

Supplementary Table 2

Supplementary Material
