# Supplementary material for: Recombination within the Cepaea nemoralis supergene is confounded by incomplete penetrance and epistasis
Source: Heredity (Edinb). 2019 Feb 14;123(2):153–61. doi: 10.1038/s41437-019-0190-6 (PMC6781172; doi:10.1038/s41437-019-0190-6)
Supplement: Supplementary file 3 — Supplementary Material [file 41437_2019_190_MOESM3_ESM.pdf]

UNIVERSITY OF LONDON, UNIVERSITY COLLEGE

GOWER STREET, LONDON, W.C.1

Telephone: Museum 7389

GALTON LABORATORY

20 April 1934.

Dear Diver,

Many thanks for the new grouse locust, which arrived in good health, though lacking one leg. I will arrange for her marriage.

In classifying some snail broods today, I found one giving apparently about 20% of crossing over. The double heterozygote was a brown bandless from Sutton Coldfield, and the double recessive from Port Isaac, Cornwall, whence I have also had bandless browns. The demonstration of crossing-over is not perfect, as the previous history of the parents is not fully known, but in the previous year the recessive had been with another brown bandless from Cornwall and the eight young had shown 3 of the 4 types to be expected if linkage was incomplete.

It is, therefore, difficult to imagine any system of previous matings which would ~~make~~ mimic the effects of ~~the~~ crossing-over in the way the 1933 brood has done, and I should much like to know if anything of the same kind has come to your knowledge in Nemoralis, - also if you have any data which show that in this species these factors are in other cases closely linked.

Yours sincerely,

R. A. Fisher

40 Pembroke Square,
Kensington, W. 8.

22 April 1934.

Dear Fisher,

I am glad the lady arrived safely, she was without her leg when I first met her.

Stelfox' experiments with brown in nemoralis are none of them very clear. I will look up the data when I get thechance and let you know the results. As far as my memory goes, I think I was forced to decide that brown was not allelomorphic to yellow and pink but something different. Looking through hastily for examples from natural populations in nemoralis I find two Worcestershire populations that give

	Browns	Pinks & yellows	Browns	P & Ys
00000	9	3	13	-
Banded	-	351	-	59

In hortensis there are quite [illegible] number of small population samples that are in the same sense. An example from Leeds gives

	B	Y
0	34	-
Bd	9	387

added to this there were 5 whites of which 2 were unbanded and 3 had the ususal hyaline bands. Another from Wilts

	B	Y
0	20	3
Bd	-	75

and one white with hyaline bands

If one or both of the parents were carrying old sperm and <u>if</u> the sperm can be held from one season to another you might get the four types. I was faced with similar difficul[illegible] in one or two of Stelfox' crosses. Yrs C.D.

UNIVERSITY OF LONDON, UNIVERSITY COLLEGE

GOWER STREET, LONDON, W.C.1

Telephone : Museum 7389

GALTON LABORATORY

24 April 1934.

Dear Diver,

Many thanks for your ~~labour~~ letter, which shows that the samples from wild populations have, in some cases, browns tightly linked with bandlessness, for even low cross-over frequencies should , in time, equalise the proportions. As to previous matings, we are only concerned with the double recessive, since previous matings of the double heterozygote could not produce the cross-over types, without upsetting the gene ratios. These are quite normal, as you will see from a letter to Nature that I have drafted and to which you might care to suggest any amendments that might occur to you.

My double recessive shared a cage, in a previous year, with another brown banded, and they produced a very small brood, only 8 being classified, but showing 3 of the 4 types . In the light of the 1933 brood, I should guess that here, also, there was crossing-over in 3 cases out of 8, and that the first brown bandless was also a double heterozygote in coupling, but at first I could not rely on this, as they had only been isolated a few months. The 1933 brood is thus on fairly strong ground, which is not weakened by previous experience, but is not absolutely

critical. I have at once put up two pairs of brown bandless in repulsion, bred in my cages, back-crossed to banded yellow, but I may not get any confirmation for 2years, so ,I think I ought to put the current result on record.

Would you care to add your wild ~~computation~~ population data and make a joint letter?

Yours sincerely,

R. A. Fisher

40 Pembroke Square,

Kensington, W. 8.

2 May 1934.

Dear Fisher,

I attach two small amendments to your wording and a proposed form for the last new para. I attach considerable importance to leaving out the word "wild" as the word is definitely misleading - all the genes concerned are wild.

In the last para, I have adhered to your first draft except for quite small changes. It does not, I think, lengthen the original letter unduly. I have given the ~~xxx~~ reference to my 1933 note as that ~~xxxx~~ sets out briefly the facts about natural populations. If we included the actual figures for the Bundoran samples that I found they would require several sentences of explanation and I do not think much would gained.

Yours sincerely,

CD.

I don't know whether you are asking for reprints of this note, but I should like a few.

CD

Proposed Amendments to the Note

"Crossing-over in the Land Snail _Cepaea nemoralis_ L.

Page 1, line 5:

Leave out "wild" and insert "normal".

line 16:

Leave out "I have" and insert "one of us (R.A.F.) has".

Page 2, at end add new para:

"The occurrence of close linkage between a number of genes in natural populations of _Cepaea_ has long been recognised though the actual data have not yet been published. ~~An examination of~~ The unpublished genetic results of A.W.Stelfox, kindly placed at the disposal of one of us (C.D.), ~~shows~~ include a similar back-cross between pink bandless and yellow banded which gave 13 pink bandless and 11 yellow banded, followed by a brood from bandless pinks of 16 pink bandless and 10 yellow banded. Neither of these progenies show recombination and they are inconsistent with the occurrence of so much as 20 per cent. It seems likely that the pinks used in the two sets of experiments were genetically different, and that the one is more closely linked with bandless while the other shows appreciable recombination. The alternative that the same genes may show ~~different~~ variable linkage in different strains is, however, ~~by no means~~ not excluded.

Footnote:- Diver, C.(1933) Proc. Sixth International Cong: Gen: Vol 2, p. 236.

C.DIVER.

40 Pembroke Square W.8

2 May 1934.

in Delhi

A.W. Shilfer has made a number of experiments with this species and he has kindly placed his results at our disposal. In one of them ~~a strong linkage~~ there is good evidence for a strong linkage between the same two pairs of genes but ~~it is not~~ the possibility is not excluded that the pinks used in the two cases were not the product of the same gene. In ~~other~~ crosses involving other genes his results show that some crossing over takes place.

138
45
183

179
14
193

Brown	Pink	Yellow
O	183	193.
Bd	127.	25
	47.	20
	309.	57

38/23 –

	Brown	Pink	yellow
O	7	3	–
Bd.	–	29	33

Eloff.
Genetica
1932. Vol XIV.
parts 1–2

a detailed exhibition of everything known about crossing over

1

The occurrence of close linkage ~~between a number of genes~~ between a number of genes in natural populations of Cepaea has long been recognised[1] ~~though~~ ~~the extensive population studies~~ ~~though the~~ though the actual ~~[illegible]~~ data have not yet been published. An examination ~~of the unpublished data~~ of the unpublished genetic ~~data~~ results obtained by A.W. Stelfox and kindly placed at the disposal of one of us (C.D.) shows ~~the~~ ~~following backcross bred from~~ a backcross between a pink bandless × yellow banded which gave 13 pink bandless and 11 yellow banded, followed by a second from bandless pinks of 16 pink bandless and 10 yellow banded. Both of these progenies show no recombination and are inconsistent with the occurrence of as much as 20%. It seems likely that the pinks used in the two sets of experiments were genetically different and that while one is more closely linked with bandless the other shows appreciable recombination. The alternative that the same genes may show variable linkage in different strains is, however, not excluded.

[1] Diver 1932

Although the general occurrence of close linkage in Cepaea has been long recognised, published evidence is lacking. One of us (C.D.) on ~~[illegible]~~ examining the unpublished data of A.W. Stelfox on Nemoralis has found ~~[illegible]~~ ~~F_2 [illegible] 20%~~ the following backcross:

P 13 Pink bandless

11 Yellow banded

followed by an F_2 from the bandless [illegible] giving

16 Pink bandless

10 Yellow banded

Both of these progenies ~~[illegible]~~ show no ~~[illegible]~~ recombination and are consistent with the occurrence of as much as 20%. It is to be presumed therefore that the [illegible] [illegible] genetically different, and that which one is more closely linked with banding, the other shows apparent recombination. The alternative that the same genes show variable linkage in different families is, however, by no means excluded.

CROSSING - OVER IN ~~CEPEA~~ the Land Snail, Cepea Nemoralis.

A genetic situation of particular interest from the point of view of the evolutionary modification of genetic phenomena occurs, not only among fishes (Lebistes), but among insects (Orthoptera) and land snails (Cepea), in all of which a number of variant genes, completely dominant to their ~~wild~~ normal allelomorph, are found to be closely linked in the same linkage group. In the ~~grass~~ grouse locusts the linkage was at first regarded as absolute and the variant forms supposed to belong to a single allelomorphic series. The occurence of crossing-over was demonstrated by Haldane in the extensive data published by Nabours. Nabours has since shown that in one American species of ~~grass~~ grouse locust (Acrydium arenosum) a similar series of pattern factors occurs, showing only relatively loose linkage. In the course of experiments designed to test quite other consequences of natural selection, ~~I have~~ One of us (RAF) has recently obtained a brood of Cepea nemoralis showing apparently 20-25% recombination between the factor for brown ~~versus~~ yellow and that for bandless. The brood of about 60 young appeared in the summer of 1933, the parents having been taken in nature about 15 and 28 months previously. The brown bandless double heterozygote was evidently in the coupling phase, for there survived to be classified in April of this year 17 bandless brown, 18

banded yellow or 35 of the parental combinations, together with 4 banded brown and 5 bandless yellow, making 9 recombinations evidently due to crossing-over.
